# Global Analysis of Nutritional Factors and Cardiovascular Risk: Insights from Worldwide Data and a Case Study in Mexican Children

**DOI:** 10.3390/jcdd12040115

**Published:** 2025-03-25

**Authors:** Karmina Sánchez-Meza, Gustavo A. Hernández-Fuentes, Estibaliz Sánchez-Meza, Ivan Delgado-Enciso, Carmen A. Sánchez-Ramírez, Roberto Muñiz-Valencia, José Guzmán-Esquivel, Idalia Garza-Veloz, Margarita L. Martinez-Fierro, Iram P. Rodriguez-Sanchez, Janet Diaz-Martinez, Joel Cerna-Cortés, Oscar F. Beas-Guzmán, Mario Ramírez-Flores

**Affiliations:** 1 Department of Molecular Medicine, School of Medicine, University of Colima, Colima 28040, Mexico; ksmeza@ucol.mx (K.S.-M.); gahfuentes@gmail.com (G.A.H.-F.); sanchez@ucol.mx (E.S.-M.); ivan_delgado_enciso@ucol.mx (I.D.-E.); carmen_sanchez@ucol.mx (C.A.S.-R.); joelcerna@ucol.mx (J.C.-C.); oscar.beas.11@gmail.com (O.F.B.-G.); 2 State Cancerology Institute of Colima, Health Services of the Mexican Social Security Institute for Welfare (IMSS-BIENESTAR), Colima 28085, Mexico; 3 Faculty of Chemical Sciences, University of Colima, Coquimatlan 28400, Mexico; rmuniz0@ucol.mx; 4 Robert Stempel College of Public Health and Social Work, Florida International University, Miami, FL 33199, USA; 5 Clinical Epidemiology Research Unit, Mexican Institute of Social Security Institute, Villa de Alvarez, Colima 28984, Mexico; jose.esquivel@imss.gob.mx; 6 Molecular Medicine Laboratory, Unidad de Medicina Humana y Ciencias de la Salud, Universidad Autónoma de Zacatecas, Zacatecas 98160, Mexico; idaliagv@uaz.edu.mx (I.G.-V.); margaritamf@uaz.edu.mx (M.L.M.-F.); 7 Molecular and Structural Physiology Laboratory, School of Biological Sciences, Universidad Autónoma de Nuevo León, San Nicolás de los Garza 66455, Mexico; iramrodriguez@gmail.com; 8 Department of Dietetics and Nutrition, Research Center in a Minority Institution, Florida International University (FIU-RCMI), Miami, FL 33199, USA; jdimarti@fiu.edu

**Keywords:** linoleic acid, cardiovascular risk, omega-6, cross-sectional study, nutrition, children

## Abstract

Cardiovascular diseases (CVDs) are the leading cause of mortality worldwide, with growing concerns about the impact of omega-6 polyunsaturated fatty acids (n-6 PUFAs) on cardiovascular health. This study aims to evaluate the relationship between serum linoleic acid (LA) levels and waist-to-height ratio (WHtR), a recognized cardiovascular risk marker, in children. The research was conducted in two parts. First, a global analysis of publicly available data (2019–2021) explored the association between nutritional factors and CVD prevalence across 183 countries. Second, a cross-sectional study involving 67 children (33 with obesity and 34 with healthy weight, classified using BMI Z-scores) examined the correlation between serum LA levels and WHtR. Global analysis revealed a moderate correlation between low seafood omega-3 fatty acid intake and CVD incidence (rho = 0.341), while low polyunsaturated fatty acid consumption showed a weak correlation (rho = 0.228). In children, a significant positive correlation was observed between serum LA levels and WHtR (rho = 0.716, *p* < 0.001), with similar correlations found when stratified by sex (girls: rho = 0.690; boys: rho = 0.709). Serum LA levels also correlated positively with weight (rho = 0.684). These findings are consistent with the existing literature, that high serum LA levels may contribute to early cardiometabolic risk in children, emphasizing the need for dietary interventions to mitigate cardiovascular risks in early life.

## 1. Introduction

Heart disease has remained the leading cause of death worldwide for the past two decades, now accounting for approximately 16% of all deaths globally. Between 2000 and 2019, deaths attributed to heart disease increased dramatically from over 2 million to nearly 9 million annually [1]. As a preventive measure, dietary guidelines have long recommended reducing saturated fat intake to less than 7% of total energy and increasing consumption of monounsaturated and polyunsaturated fatty acids (PUFAs) [2]. This led to a 54% rise in PUFA consumption between 1975 and 2005 [3]. However, recent studies have questioned these recommendations, with evidence suggesting that diets high in n-6 PUFAs may adversely impact cardiovascular health [4].

Dietary patterns play a crucial role in influencing cardiovascular health. In many Western countries, diets high in saturated fats, trans fats, and refined sugars, commonly referred to as the “Western pattern diet”, have been linked to increased risks of obesity, type 2 diabetes, and CVD [5,6]. Conversely, traditional diets in Mediterranean regions, characterized by high consumption of fruits, vegetables, whole grains, and healthy fats like olive oil, have been associated with lower cardiovascular risk [7]. The Seven Countries Study highlighted that populations adhering to such diets exhibited lower rates of heart disease [8]. In developing countries, rapid urbanization and globalization have led to a nutrition transition, shifting dietary patterns towards increased intake of processed foods, sugars, and fats [9,10]. This shift has been linked to rising obesity rates and a dual burden of malnutrition, where undernutrition and overnutrition coexist, leading to increased risks of CVD [10].

Common sources of n-6 PUFAs, particularly linoleic acid (LA), include vegetable oils such as soybean, corn, sunflower, and safflower oil. These oils are widely used in the preparation of processed and fried foods, which constitute a significant part of the modern diet [11,12]. For children, primary sources of n-6 PUFAs include snacks such as chips, cookies, pastries, and fast food, as well as commercially prepared meals. Additionally, natural sources of n-6 PUFAs, particularly linoleic acid, include seeds (such as sunflower, sesame, and pumpkin seeds), nuts (such as walnuts, almonds, and pecans), and certain plant-based oils like safflower and grapeseed oil [13,14]. While these natural sources are generally more nutrient-dense, providing essential vitamins, minerals, and antioxidants [11,12]. However, the prevalence of processed food options has led to increased consumption of less nutritious sources among younger populations, raising concerns about their potential health effects [15,16,17].

Long-chain PUFAs, particularly omega-3 (n-3) and omega-6 (n-6), have been shown to exert opposing effects. While n-3 PUFAs are recognized for their cardioprotective and anti-inflammatory properties, n-6 PUFAs, including LA, have been associated with pro-inflammatory responses [18,19]. However, this simplistic view, which categorizes omega-6 PUFAs as inherently pro-inflammatory and omega-3 PUFAs as anti-inflammatory, has been updated in recent research. Emerging evidence shows that the effects of omega-6 PUFAs depend on the context of overall dietary intake, particularly in relation to omega-3 fatty acids [20,21,22]. Although some clinical trials propose that there is a moderate benefit of n-6 PUFA consumption, predominantly LA, in reducing coronary disease risks, findings remain inconsistent. Higher blood or adipose tissue levels of LA have been linked to lower cardiovascular risks in some studies [4]. Nevertheless, a systematic review by Khandelwal et al. highlighted inconclusive evidence from both clinical trials and observational studies regarding the association between n-6 PUFA intake and cardiovascular disease (CVD) [18].

Recent meta-analyses and systematic reviews have emphasized the need to consider the balance between n-6 and n-3 PUFA intake rather than assessing n-6 PUFAs in isolation [20]. Studies consistent with the existing literature show that an excessive intake of n-6 PUFAs, particularly when not accompanied by adequate n-3 PUFA consumption, may contribute to a pro-inflammatory state that increases the risk of cardiovascular and metabolic disorders [21,22]. Emerging evidence supports the idea that a lower ratio (closer to 5:1 rather than the Western diet’s typical 20:1) is associated with reduced cardiovascular risk [23]. Despite these findings, dietary trends in many countries continue to favor high n-6 PUFA consumption, raising concerns about its long-term effects on cardiometabolic health, particularly in vulnerable populations such as children [24,25,26].

Childhood obesity has become a global epidemic, with the World Health Organization (WHO) estimating that over 39 million children under the age of 5 were overweight or obese in 2020 [27]. This increase is attributed to unhealthy diets, sedentary lifestyles, and socio-economic factors. In Mexico, childhood obesity is a critical public health issue, with the country ranking among the highest in the world for obesity rates in children. Recent data indicate that approximately 35% of Mexican children aged 5–11 years are overweight or obese, driven by high consumption of processed foods and sugary beverages combined with insufficient physical activity [16,17,28,29]. This alarming trend predisposes children to cardiometabolic risks, including type 2 diabetes and cardiovascular diseases, later in life [15]. Central obesity, measured by the waist-to-height ratio (WHtR), is considered a better predictor of cardiovascular risk than body mass index (BMI) in children [30,31]. Given the limited data on the association between n-6 PUFA intake and cardiovascular risk in children, this study consists of two main parts: first, a global analysis of publicly available data (2019–2021) exploring the association between nutritional factors and cardiovascular disease (CVD) prevalence across 183 countries; and second, an investigation into the relationship between serum linoleic acid (LA) levels and the waist-to-height ratio (WHtR) in pediatric populations. The global analysis provides a broader context for understanding nutritional influences on CVD, while the pediatric-focused portion aims to assess the potential cardiometabolic risks associated with n-6 PUFA consumption in children. Together, these analyses aim to provide a more comprehensive understanding of the impact of dietary fats on cardiovascular health worldwide and in vulnerable pediatric populations.

## 2. Materials and Methods

The methodology for this study was divided into two main sections. In the first part, an analysis of databases was conducted to examine the relationship between nutritional parameters and the prevalence of cardiovascular diseases (Section 2.1). In the second part, a study was designed to evaluate the correlation between serum linoleic acid (LA) levels and the waist-to-height ratio (WHtR), a recognized cardiovascular risk marker, in children (Section 2.2).

### 2.1. Data Collection and Selection

An extensive review of information was conducted using international and publicly accessible databases over the years 2019 to 2021. A comprehensive set of empirical variables was selected to capture a wide range of nutritional influences that may contribute to cardiovascular risk (Table 1). Data regarding these factors were systematically collected for each of the 183 countries included in our study. Table 1 provides a detailed overview of the variables used in this study. Each variable was selected based on its documented relevance to cardiovascular risk, and the data were collected from verified sources to ensure reliability and consistency across all countries. The table also highlights the specific databases from which each variable was derived, as well as the criteria used for data standardization and comparison across regions [32].

#### 2.1.1. Inclusion and Exclusion Criteria

All freely accessible online databases that provided quantifiable data and values for each of this study’s variables were included, provided they offered global information covering at least 183 countries. Studies and databases that did not provide worldwide data or lacked full accessibility were excluded from the analysis.

#### 2.1.2. Data Analysis

Quantitative data for each variable were collected for all countries included in our study, resulting in the creation of an extensive database that served as the foundation for the subsequent analysis [36]. For the dependent variable, cardiovascular prevalence, the age-standardized rate (ASR) in percent was employed. ASR is a measure commonly used in epidemiology and public health to compare disease rates across different populations, accounting for variations in age distribution within said populations. This approach enables a more reliable comparative analysis across diverse populations [37,38]. Statistical analyses were conducted concurrently using SPSS to ensure robust results [19,20]. Initially, the Kolmogorov–Smirnov test was applied to assess data normality, given the large sample size. Upon confirming that the data did not follow a normal distribution, a non-parametric approach was adopted, utilizing Spearman’s correlation to examine the relationship between all variables [39]. To address the issue of multiple comparisons and reduce the risk of false positives, the Benjamini–Hochberg false discovery rate (FDR) correction was applied [40]. This correction method was implemented for the 15 variables analyzed, ensuring that the reported associations are statistically robust while maintaining adequate power in the study [41].

#### 2.1.3. ROC Curves

To evaluate the predictive capacity for the incidence rate of cardiovascular diseases—expressed as the number of new cases per 100 individuals, age-standardized, and calculated for both sexes—the areas under the ROC curve (AUCs) were calculated for the different variables, along with their 95% confidence intervals, cut-off points, *p*-values, sensitivity, specificity, and predictive values. Predictive capacity was classified based on AUC values as follows: 0.50–0.60 (failed), 0.61–0.70 (worthless), 0.71–0.80 (poor), 0.81–0.90 (good), and >0.90 (excellent), as previously described [42,43] (54.55). The cut-off point was determined at the point on the curve that maximized sensitivity and specificity [44]. Sensitivity and specificity were classified as follows: high (>80%), moderate (65–80%), and low (<65%) [45].

#### 2.1.4. Ethical Considerations

This part of the study adheres to ethical guidelines concerning data privacy in the collection, handling, and analysis of the data [46].

#### 2.1.5. Limitations and Assumptions

Potential limitations identified in this study include the availability of data over the years, as some referenced databases do not always retain previous datasets when updating their information. Additionally, the quality and scope of the selected sociodemographic factors were considered as limiting factors. We acknowledge the assumptions made during the mathematical modeling process and their potential impact on the results.

### 2.2. Study Design

For the second part of the study, a cross-sectional study was conducted on 67 children through convenience sampling from the nutrition department of a primary care hospital. Participation was contingent upon obtaining signed informed consent from the parents or guardians, who were provided with verbal and written explanations about the study’s purpose and procedures. Prior to the inclusion of the children in the study, parents or present guardians completed a general questionnaire to gather information about the socioeconomic status (classified according to the 2022 guidelines of the Mexican Association of Market Intelligence and Opinion Agencies [AMAI], parents’ occupations, and general activities. Households were classified into three groups: A/B (high), C (middle level), and D/E (vulnerable middle class/poor) [47,48]. Data collection included measurements of height, weight, and waist circumference for all participants. The variables analyzed comprised the percentage of the area under the curve (%AUC) of LA in serum, the waist-to-hip ratio (WHR), and the body mass index (BMI). Eligibility criteria included the following: (I) children aged 60–144 months; and (II) availability of essential data, such as age, sex, BMI, and waist circumference (WC). Children with diabetes or other chronic diseases or those with incomplete anthropometric data were excluded from the study. The sample size of 67 participants was determined using a formula for comparing two independent means. Since most of the participants were ambulatory patients, dietary control was not implemented.

### 2.3. Anthropometric and Body Composition Assessments

All measurements were performed by trained technicians. Prior to data collection, the lead author and two collaborators conducted an anthropometric standardization trial to evaluate both consistency (intra-group individual measurements) and validity (inter-group comparison against a gold standard). The anthropometric technique was refined and adjusted until the desired intra- and inter-group correlations were achieved. For weight and height measurements, a digital scale equipped with a stadiometer measuring 64–214 cm in length (TANITA WB-3000) was used.

Measurements were taken following standard protocols, with children wearing lightweight clothing and no shoes [49]. BMI was calculated as weight (kg) divided by height squared (m^2^). Obesity and healthy weight categories for the children were defined according to the age-specific BMI Z-scores established by the WHO. Children with BMI Z-scores ≥ +2 SD were classified as obese, while those with scores between ≥2 SD and +1 SD were classified as having a healthy weight. Waist circumference (WC) was measured using a fiberglass tape positioned above the uppermost lateral border of the right ilium. All measurements were recorded to the nearest millimeter at the end of a normal expiration, with children standing upright, feet together, and arms relaxed at their sides. Cardiometabolic risk was assessed using the waist-to-height ratio (WHR), calculated by dividing height (cm) by WC (cm), with a WHR ≥ 0.50 indicating increased risk.

### 2.4. Linoleic Acid (LA) Determination

A 12-h fasting blood sample was collected via venipuncture. Serum was obtained and stored in labeled tubes at −75 °C for subsequent fatty acid analysis. Linoleic acid (LA) was extracted using the Folch method [50,51], which involves a CHCl_3_:MeOH solution (2:1, *v*/*v*). The extracted samples were analyzed through gas chromatography coupled with mass spectrometry (GC-MS) using a Varian 3900 gas chromatograph paired with a Saturn 2100T mass spectrometer detector (Varian, Palo Alto, CA, USA). An Omegawax 320 capillary column (30 m × 0.32 mm × 0.25 µm) was utilized for the analysis. Identification of LA was achieved by comparing retention times with those of a standard LA mixture (Sigma-Aldrich, St. Louis, MO, USA). Quantification was performed by measuring the area under the peak (AUC) and comparing it to the LA standard. Results were reported as the percentage of the AUC (%AUC). The chromatographic peaks for LA were calculated for each child, although these data are not displayed.

### 2.5. Statistical Analysis

Data were analyzed using the SPSS software, version 20 [39]. The Kolmogorov–Smirnov test was performed, and the data had not parametric distribution [52]. The variables were described as frequencies and percentages when qualitative and as median and interquartile range when quantitative. Inferential statistics were carried out using the U de Mann–Whitney test and Spearman correlation. Statistical significance was set at a *p*-value < 0.05 [53].

### 2.6. Ethical Considerations

The protocol was approved by the Ethics Committee of the Hospital General Zona 1 IMSS (Registration Number: R-2014-601-9), in accordance with the principles outlined in the Declaration of Helsinki, including beneficence, non-maleficence, justice, and respect for autonomy. Informed consent was obtained from the legal guardians of the minors involved in the study [54].

## 3. Results

In the first part of our study, we analyzed data from 183 countries, correlating them with 14 potentially associated variables. Initial normality tests indicated that many of the variables did not follow a normal distribution. As a result, we applied non-parametric statistical methods to analyze the data, which are more appropriate for distributions that deviate from normality.

**Figure 1:**
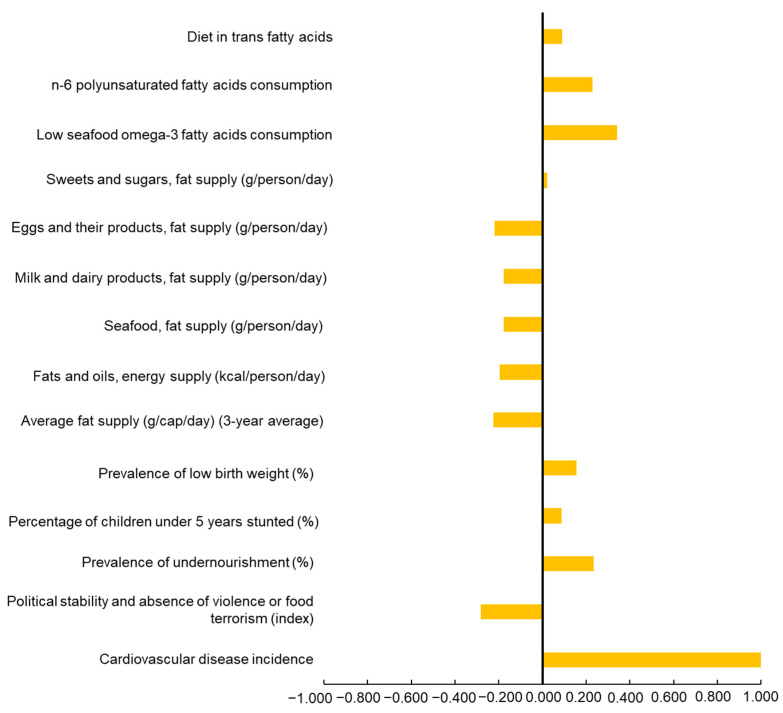
Spearman’s Rho correlation analysis of nutritional variables versus cardiovascular disease incidence. Negative values indicate inverse correlations, while positive values indicate direct correlations. The Benjamini–Hochberg false discovery rate (FDR) correction was applied to adjust for multiple comparisons across the 15 variables analyzed, ensuring statistical robustness and minimizing false positives.

The correlation analysis was performed between global variables and cardiovascular risk, measured as the incidence of cardiovascular diseases per 100 people (age-standardized, in both sexes). A total of 15 variables were analyzed (Figure 1). First, the analysis revealed a moderate negative correlation between political stability and absence of violence (−0.281), showing an association between greater instability may increase cardiovascular risk, potentially due to stress and limited access to healthcare in such environments. Second, a positive correlation with undernourishment prevalence (0.233) was observed, indicating that chronic nutritional deficiencies may exacerbate cardiovascular risk. Other health-related variables, such as low birth weight (0.156) and stunted growth in children (0.086), showed weaker positive correlations, reflecting the long-term impact of early-life health conditions.

Third, dietary factors showed notable trends. Fats from fish (−0.177), milk (−0.178), and eggs (−0.221) had negative correlations, showing an association with a protective effect against cardiovascular risk. Conversely, diets low in seafood omega-3 fatty acids (0.341) and polyunsaturated fatty acids (0.228) exhibited strong positive correlations with cardiovascular risk. It is important to mention that the variable diet low in polyunsaturated fatty acids is defined as the average daily consumption (in percentage of daily energy) of less than 9–10% of total energy intake from omega-6 fatty acids, specifically including linoleic acid, γ-linolenic acid, eicosadienoic acid, dihomo-γ-linolenic acid, and arachidonic acid. These findings highlight the critical role of linoleic acid and its derivatives in cardiovascular health.

In the second part of this global analysis, focused on the consumption of specific dietary fats, caloric contributions, and nutritional stability, we conducted a predictive evaluation of certain variables to assess their capacity to predict cardiovascular risk. Variables were selected based on their Spearman correlation strength and statistical significance, prioritizing those with higher discriminatory capacity (Table 2). The results revealed the following key findings:

First, low seafood omega-3 fatty acid consumption emerged as the strongest predictor, with an AUC of 0.676, high sensitivity (89.30%), and strong statistical significance (*p* < 0.0001). While its specificity was moderate (53.80%), its high sensitivity and a strong negative predictive value (82.70%) make it a reliable indicator for identifying individuals at risk. This aligns with its positive Spearman correlation (0.341), but the practical implications of this association for cardiovascular risk assessment require further investigation in longitudinal studies.

Second, the prevalence of undernourishment (%) also demonstrated strong predictive capacity, with an AUC of 0.675, high sensitivity (87.50%), and adequate specificity (57.30%). Its positive predictive value (68.60%) and negative predictive value (81.10%) further support its reliability. The strong Spearman correlation (0.233) confirms the significance of this variable as an independent predictor, emphasizing the importance of addressing nutritional deficiencies to mitigate cardiovascular risk. Third, the diet low in polyunsaturated fatty acids, which include linoleic acid and its derivatives, showed promising predictive capacity with an AUC of 0.650 and strong statistical significance (*p* < 0.0001). It exhibited high sensitivity (86.70%) but limited specificity (43.20%), making it particularly useful for identifying positive cases. This result aligns with the Spearman correlation (0.228), highlighting the relevance of polyunsaturated fatty acids, including linoleic acid, in cardiovascular health.

In contrast, other variables showed limited predictive utility. For example, low birth weight prevalence (%) displayed a low AUC of 0.606, moderate sensitivity (65.80%), and low specificity (58.90%), constraining its practical application despite its statistical significance (*p* = 0.0270). Similarly, average fat supply (g/cap/day) had poor discriminatory capacity with an AUC of 0.409, moderate sensitivity (61.00%), and high specificity (87.80%), limiting its overall relevance.

Variables such as diets high in trans fatty acids (AUC 0.462) and political stability and absence of violence (AUC 0.351) demonstrated no meaningful discriminatory capacity, despite some achieving statistical significance. Their low AUC values nullify their practical predictive value. This predictive analysis identifies low seafood omega-3 fatty acid consumption and prevalence of undernourishment (%) as the most reliable global predictors of cardiovascular risk due to their strong discriminatory capacity, high statistical significance, and robust predictive values. Additionally, the role of polyunsaturated fatty acids, particularly linoleic acid, is underscored by their predictive strength.

**Figure 2:**
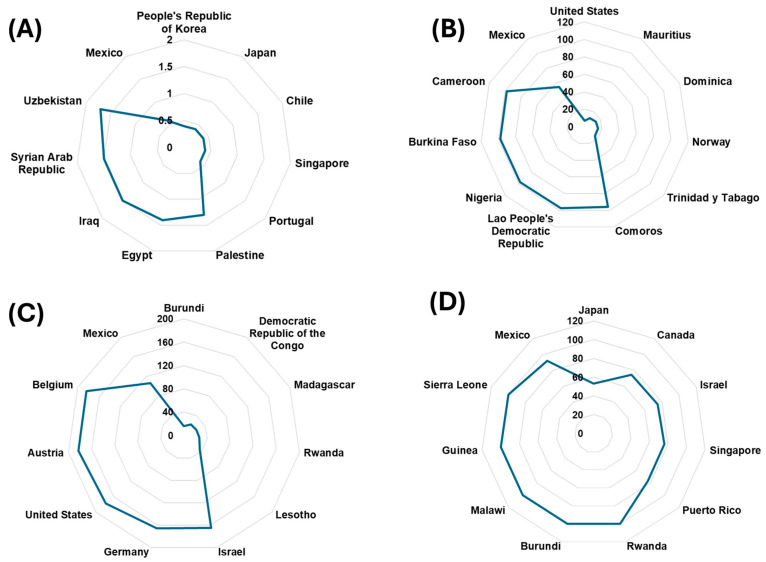
Comparative analysis of four variables related to cardiovascular health across countries. (**A**). Cardiovascular disease incidence. (**B**). Polyunsaturated fatty acid consumption. (**C**). Average fat supply (g/cap/day) (3-year average). (**D**). Low seafood omega-3 fatty acid consumption. The data compare the five countries with the highest levels and five countries with the lowest levels of each variable, alongside Mexico, for each respective category.

Based on the analysis of the four selected variables, a comparative evaluation was conducted between countries representing the extremes of each variable and Mexico, positioned as an intermediate case (Figure 2). The findings offer valuable insights: For the average fat supply (g/capita/day), countries at the lower end, such as Burundi (15.9 g/capita/day) and the Democratic Republic of the Congo (22.8 g/capita/day), demonstrate a significantly limited availability of dietary fats compared to Mexico, which registers 106.6 g/capita/day. In contrast, countries like Germany (166.1 g/capita/day) and Belgium (183.6 g/capita/day) are positioned at the higher end, reflecting diets richer in fats. Mexico’s intermediate position likely reflects a dietary pattern that includes a combination of traditional and modern eating habits.

Regarding n-6 polyunsaturated fatty acid consumption, Mexico’s value (53.87 g/capita/day) places it closer to countries with higher intake, contrasting with countries like the United States (6.76 g/capita/day), indicating a relatively insufficient consumption of these beneficial fatty acids in Mexico. Conversely, countries like Cameroon (97.87 g/capita/day) and Burkina Faso (97.33 g/capita/day) rank among the highest, indicating healthier dietary patterns with higher levels of polyunsaturated fats. In terms of the number of new cases of cardiovascular diseases per 100 people (age-standardized), Mexico records a value of 0.6%, which is higher than countries such as Japan and Chile, both of which report 0.4%. These differences may reflect variations in dietary habits, physical activity, and access to healthcare systems among these nations.

Finally, for the variable diet low in omega-3 from seafood sources, Mexico exhibits a high percentage (91.92%) of the population with inadequate consumption. This unfavorable position aligns it with countries such as Canada (74.29%) and Israel (75.11%). In stark contrast, Japan stands out with the lowest percentage (53.37%), likely due to its traditional diet rich in fish and seafood. These findings highlight the nutritional challenges faced by Mexico, particularly the high prevalence of inadequate seafood-derived omega-3 consumption and relatively low polyunsaturated fatty acid intake. These factors are likely contributors to the observed cardiovascular risk in the population. This context underscores the urgent need for targeted nutritional interventions aimed at promoting the consumption of healthier fats and improving dietary patterns to reduce cardiovascular risk.

Our analysis highlights an association between linoleic acid (LA) and cardiovascular health, which warrants further investigation to explore the potential underlying mechanisms. We observed that diets low in polyunsaturated fatty acids, particularly those rich in LA, were strongly correlated with an increased risk of cardiovascular diseases. These findings indicate an association between linoleic acid, along with its derivatives intake, and cardiovascular risk markers cardiovascular risk. Based on this, it is crucial to promote dietary changes aimed at increasing the intake of LA and other polyunsaturated fats as a strategy to reduce cardiovascular risk and improve overall heart health.

In order to establish the relationship between the global variables observed, such as the incidence of cardiovascular diseases and polyunsaturated fatty acid consumption, in a specific context, the analysis was conducted in a sample of Mexican children. The study population consisted of 67 children from Villa de Álvarez, Colima, who attend medical consultations. According to the socioeconomic classification ABCDE (specification for 2021), 65% of the children belong to levels D–E (lower middle class—lower class), 20% to level C (middle class), and 15% to levels A–B (upper middle class—upper class). Regarding the parents’ occupation, in 60% of the households, the mother is exclusively dedicated to the home. The remaining mothers are employed by the government (27%), 7% are either employees or business owners, and the rest are engaged in other activities. As for the male heads of households, 68% work in government sectors (public education), 19% are involved in commerce, and 13% are dedicated to agriculture or livestock. Regarding access to healthcare services, 80% of the children come from families covered by the IMSS (Mexican Institute for Social Security) or other public health services, while the remaining 20% do not have social security. Concerning participation in extracurricular activities, 90% of the children do not engage in organized activities outside of school, such as sports or recreational classes.

The mean age of 105 ± 26 months. Of these, 29 (43.3%) were girls, of whom 15 had a healthy weight and 14 were classified as obese. The remaining 38 children (56.7%) were boys, with 19 having a healthy weight and 19 classified as obese. The demographic and anthropometric data, stratified by sex and body weight, are detailed in Table 3.

Cardiovascular risk was observed in 51.7% (15/29) of the girls and 57.8% (22/38) of the boys, with no significant difference between the sexes (*p* = 0.135). Among children with a healthy weight, cardiovascular risk was present in 14.7% (5/34), whereas it was significantly more frequent in obese children at 96.9% (32/33).

**Figure 3:**
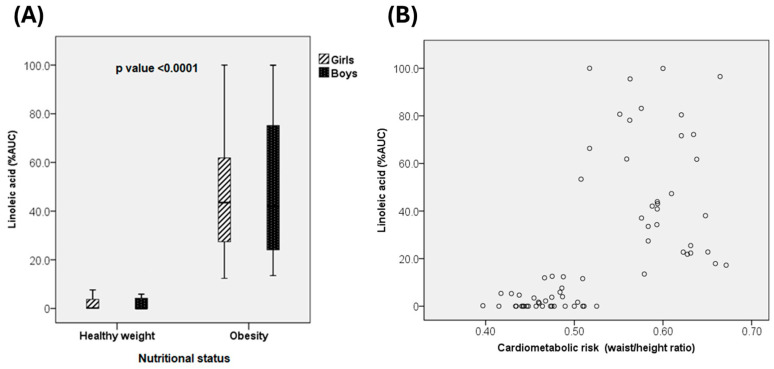
(**A**) Comparison of plasma linoleic acid levels according to nutritional status (healthy weight vs. obesity) and sex (girls vs. boys). (**B**) Correlation between plasma linoleic acid levels and cardiometabolic risk markers in school children.

When comparing linoleic acid (LA) levels between children with healthy weight and those with obesity, a significant difference was observed, with higher LA levels in the obese group (Figure 3A).

A moderate, positive, and significant correlation was found between LA levels and the waist-to-height ratio (WHtR) across the entire study population (rho = 0.716, *p* < 0.001) (Figure 3B). Sex-stratified analysis revealed similar significant positive correlations (*p* < 0.001) in both girls (rho = 0.690) and boys (rho = 0.709). Additional significant positive correlations were identified between LA levels and waist circumference (rho = 0.692, *p* < 0.001), as well as weight (rho = 0.684, *p* < 0.001).

## 4. Discussion

In this study, Spearman correlation analysis of global variables associated with cardiovascular risk revealed key findings. A moderate negative correlation was found between political stability and cardiovascular risk (−0.281), suggesting that instability may increase risk due to stress and limited healthcare access. Additionally, a positive correlation with undernourishment prevalence (0.233) highlighted the exacerbating effect of nutritional deficiencies. Dietary factors also played a significant role: consumption of fats from fish, milk, and eggs negatively correlated with risk, while low intake of omega-3 and polyunsaturated fatty acids was positively correlated, emphasizing the harmful impact of insufficient intake of these nutrients.

The predictive analysis identified low seafood omega-3 consumption and undernourishment prevalence as strong predictors of cardiovascular risk, with sensitivities of 89.3% and 87.5%, respectively, and AUC values of 0.676 and 0.675. In contrast, political instability, a diet high in trans fatty acids, and low birth weight, while statistically significant, showed limited predictive value due to low AUCs. Mexico’s intermediate position in fat supply and polyunsaturated fat intake, particularly in omega-3 consumption, underscores the need for targeted nutritional interventions to improve heart health, especially by increasing omega-3 and polyunsaturated fat intake.

With respect to the second part of the study, this is the first study that describes an association between the levels of linoleic acid (LA) and waist-to-hip ratio (WHR) as a predictor of cardiovascular disease (CVD) risk in children. The result of our study shows an association between higher levels of LA and higher WHR in children of both sexes, indicating a potential cardiovascular risk in this population. This association is particularly important given the growing concerns about the effects of omega-6 polyunsaturated fatty acids (PUFAs), such as LA, on CVD. The literature presents a debate about the role of omega-6 PUFAs in CVD. A study involving 42 men with coronary artery disease and 40 men without the disease found that LA and arachidonic acid (ARA) levels were significantly higher in the coronary artery disease group, showing that higher levels of these fatty acids may contribute to LDL oxidation and the formation of atherosclerotic plaques, both of which increase CVD risk [49].

Some studies employing animals have shown that increased intake of LA over five weeks was found to elevate left ventricular mass and rigidity, further enhancing the risk of cardiovascular events [4]. However, other studies have reported contrasting findings. Some have found no associations between circulating LA levels and atherosclerosis, and in certain cases, LA supplementation has been shown to reduce the inflammatory response, particularly in the heart, by decreasing triglyceride levels [55,56]. The evidence, therefore, remains mixed, highlighting the need for more research into the impact of LA on cardiovascular health, particularly across different age groups and metabolic conditions. While findings in animal models suggest a possible association between omega-6 fatty acids, such as linoleic acid (LA), and cardiovascular risk, it is crucial to recognize that conclusions derived from animal models cannot be directly extrapolated to humans. Human studies present varied results, emphasizing the need for further research to better understand the impact of LA on cardiovascular health, particularly in the pediatric population.

In humans, few studies have specifically examined the relationship between LA and cardiovascular risk in pediatric populations, although some insights can be drawn from research in adults. For instance, in middle-aged women, higher circulating levels of LA were associated with improved cardiovascular risk markers, including lower LDL cholesterol and reduced inflammatory markers. Conversely, in elderly individuals with preexisting metabolic conditions such as diabetes and hypertension, elevated LA levels were linked to increased arterial stiffness and oxidative stress, factors that contribute to CVD risk [57]. In adolescents, studies have shown that diets high in omega-6 fatty acids, including LA, correlate with increased central adiposity, a key risk factor for cardiovascular disease [14,15,58]. Contrarily, an analysis of 30 cohort studies concluded that higher LA levels were significantly associated with a lower risk of total CVD, cardiovascular mortality, and ischemic stroke [59]. Moreover, a systematic review of n-6 PUFAs concluded that there was no clear association between these fats and CVD risk, emphasizing the inconclusive nature of current evidence [18]. A recent review shows that LA consumption can reduce lipid risk markers for CVD in healthy individuals, although it did not account for age restrictions or obesity status [13]. Despite some contradictions in previous studies, our results underscore the importance of monitoring and balancing the intake of omega-6 and omega-3 fatty acids, positioning them as a key risk marker, especially in children, to prevent long-term cardiovascular risks.

It is important to highlight that in the typical Western diet, which is high in processed foods, fried foods, and oils rich in omega-6 fatty acids like LA, the imbalance between omega-6 and omega-3 fatty acids is a significant concern [60]. This imbalance, characterized by excessive omega-6 intake and insufficient omega-3 intake, has been linked to chronic inflammation, which in turn increases the risk of metabolic disorders such as obesity, insulin resistance, and hypertension—factors strongly associated with the development of cardiovascular diseases. Conversely, diets rich in omega-3 fatty acids, found in foods such as fatty fish, flaxseeds, and walnuts, may help counteract the pro-inflammatory effects of omega-6 fatty acids and reduce CVD risk. Therefore, shifting toward a more balanced intake of omega-3 and omega-6 fatty acids could be an effective strategy in mitigating cardiovascular and metabolic diseases [61,62].

In Mexico, the typical diet similarly emphasizes processed foods, fried foods, and oils rich in omega-6 fatty acids, particularly in the use of vegetable oils for cooking and the widespread consumption of fried snacks and fast food [16,17]. This dietary pattern, similar to that of the Western diet, can lead to an increased risk of cardiovascular diseases when omega-6 intake is not balanced by omega-3 intake. Although the traditional Mexican diet includes a variety of fruits, vegetables, and legumes, it often lacks adequate sources of omega-3 fatty acids, such as fish, chia seeds, and walnuts. The resulting imbalance between omega-6 and omega-3 fatty acids is a major factor contributing to the high prevalence of cardiovascular diseases in Mexico [2,56,63]. To mitigate this, promoting a balanced diet with increased omega-3 intake and reduced reliance on omega-6-rich processed foods could help reduce the cardiovascular disease burden in the Mexican population.

Previous studies like some meta-analyses have shown that body weight is associated with alterations in fatty acid composition, particularly omega-6 PUFAs [20,64]. In our study, we observed a positive and significant correlation between the children’s weight and levels of LA. This finding highlights the importance of considering body weight when evaluating the relationship between omega-6 fatty acids and cardiovascular risk in children. In our study the use of WHR as a measure of cardiovascular risk because studies in pediatric populations have demonstrated its utility as a predictor of other cardiovascular risk factors, such as cholesterol levels, blood pressure, and lipid profiles, compared to other measures such as BMI and waist circumference [65]. Furthermore, some studies indicated that compared to fasting blood lipids and glucose panels, anthropometric markers like waist circumference and WHR may not be the ideal indicators of metabolic comorbidities in obese children and adolescents [66]. Other studies employing pediatric and adult populations have found that individuals with higher WHR tend to have higher left ventricular mass, showing that a WHR < 0.5 is ideal for lowering the risk of cardiovascular disease, especially in adults [67,68]. Additionally, some advantages are that WHR is more convenient than WC alone for clinical use, as it does not require age, gender, and race reference points [69], being useful for both sexes and all groups of ages [70]. Waist circumference to height ratio, a measure of central obesity, is a better predictor of cardiovascular risk than body mass index in the pediatric population.

Socio-economic factors play a critical role in shaping dietary patterns and influencing cardiovascular risk, particularly in both Mexico and Western countries. In regions with limited access to healthy foods, individuals are more likely to rely on inexpensive, processed foods that are often high in omega-6 fatty acids and low in omega-3s. This dietary pattern can exacerbate the imbalance between omega-6 and omega-3 fatty acids, which has been linked to increased inflammation and cardiovascular risk. In Mexico, where many low-income populations face barriers to accessing nutritious foods, such as fresh fish and other omega-3-rich sources, the overconsumption of processed foods like tortillas, fried snacks, and fast foods becomes more common. In Western countries, socio-economic disparities also contribute to poor dietary choices, where access to affordable, healthy food is often limited in lower-income neighborhoods, exacerbating the risk of obesity and cardiovascular diseases (CVDs) [6,71]. Addressing these socio-economic disparities requires a multi-faceted approach, particularly in public health policy and nutrition education. Policymakers need to prioritize making healthier food options more accessible and affordable, especially for disadvantaged populations. Interventions could include subsidies for omega-3-rich foods, increased availability of fresh produce in food deserts, and educational campaigns that promote balanced diets. Furthermore, nutrition education programs should focus on the importance of balanced omega-6 to omega-3 intake and the impact of diet on long-term health [72,73]. By emphasizing practical, culturally appropriate dietary changes and ensuring that vulnerable populations are equipped with the knowledge to make healthier food choices, these interventions can help reduce the burden of CVD.

This study has several limitations. In regard to the global analysis, First, it is important to note that this study is cross-sectional in design, and while associations are observed, it is not possible to infer causal relationships between these variables. Second, the use of secondary data from public databases introduces heterogeneity due to differences in data collection methodologies across countries. Despite efforts to use recent data under consistent conditions, many countries lack standardized reporting, which may affect the robustness of cross-country comparisons. Additionally, while some confounders were controlled for, lifestyle factors such as physical activity and dietary habits were not comprehensively addressed, potentially leading to residual confounding. Finally, the low/moderate predictive value for some variables, such as polyunsaturated fatty acid consumption, may be linked to the limitations of the data sources, emphasizing the need for more standardized and updated information sharing across countries. Regarding the second part of this study, it is important to highlight that since this study represents an initial approach, future research should consider incorporating additional variables, such as dietary intake frequency, lifestyle habits of both children and parents, and other factors influencing nutritional status in order to obtain more information. Also, while LA was measured in serum in this study, the gold standard for measuring fatty acid levels is through erythrocytes. However, PUFAs measured in serum have been shown to correlate well with recent intake, especially over the past two weeks [74,75]. Furthermore, another limitation is the lack of data on LA intake through dietary surveys and other lifestyle factors, as well as the relatively small sample size, are key limitations.

The findings of this study highlight the need for public health campaigns and clinical guidelines to incorporate dietary recommendations that focus on balancing omega-6 and omega-3 fatty acids, especially in children who are in the early stages of developing cardiometabolic risk. Healthcare providers should be equipped with the tools to educate parents and caregivers about the potential risks of unbalanced fatty acid consumption, as well as the importance of nutrient-dense foods in promoting heart health from an early age. This could involve integrating dietary counseling into routine pediatric care, where healthcare providers can assess dietary patterns and offer personalized guidance. Additionally, national public health strategies should aim to address the root causes of poor dietary patterns, such as socio-economic inequality, to create an environment where healthy eating is not only possible but also encouraged.

Finally, an important point to highlight is that the proportion of linoleic acid (LA) in various foods varies significantly, with high levels typically found in vegetable oils such as sunflower, corn, and soybean oil, as well as in processed products that use these oils as ingredients. However, the exact LA content in many commercially available products is often not explicitly labeled, making it challenging for consumers to monitor their intake. A useful strategy would involve improving food labeling to include the LA content, alongside educating the public about its appropriate consumption levels. Additionally, a lack of knowledge regarding the correct use and cooking temperatures of certain LA-rich foods can exacerbate risks. For example, heating oils containing high levels of LA at excessive temperatures can lead to the formation of toxic compounds, such as aldehydes and trans fats, which may increase oxidative stress and inflammation, compounding the inherent risks associated with LA consumption. Public health campaigns emphasizing these aspects could help mitigate the potential adverse effects while promoting a more balanced dietary approach.

## 5. Conclusions

In conclusion, our study shows a significant association between higher serum levels of linoleic acid (LA) and cardiovascular risk, as indicated by the waist-to-hip ratio (WHR) in children. This finding points to the potential impact of omega-6 fatty acids on cardiometabolic risk in early life stages, highlighting the importance of maintaining a balanced intake of fatty acids. While there remains ongoing debate about the exact role of omega-6 polyunsaturated fatty acids (PUFAs) in cardiovascular health, promoting a balanced diet with appropriate levels of these fatty acids could be a useful consideration in preventive strategies for cardiovascular diseases.

Moreover, our study underscores the relevance of global variables, such as political stability, prevalence of undernourishment, and dietary patterns, which were found to correlate with cardiovascular risk. These findings are consistent with the existing literature, which has identified an association in different populations worldwide, and the importance of dietary factors, including omega-6 and omega-3 fatty acids, could be extrapolated to other regions, particularly in areas with high rates of undernourishment or political instability. Given the complex relationship between diet, socio-economic factors, and cardiovascular risk, further well-designed studies are needed to better understand the effects of dietary fatty acids on cardiovascular health in children and to assess the applicability of these findings across diverse global contexts.

## Figures and Tables

**Table 1:** Sociodemographic factors studied from each country.

Variables	Description	Reference
Cardiovascular disease incidence	Number of new cases of cardiovascular diseases per 100 people, in both sexes, age-standardized percent (%).	Our World in Data [33]
Political stability and absence of violence or food terrorism (index)	A situation where all people, at all times, have physical, social, and economic access to sufficient, safe, and nutritious food that meets their dietary needs and food preferences for an active and healthy life.	Food and Agriculture Organization of the United Nations (FAO) [34]
Prevalence of undernourishment (%) (3-year average)	A proportion of the population is in a state of undernourishment. Undernourishment is defined as the condition of people whose food energy consumption is consistently below the minimum food energy requirements for leading a healthy life and engaging in light physical activity.	
Percentage of children under 5 years stunted (modeled estimate) (%)	Proportion of children under 59 months whose height-for-age is below the median of the reference population adopted by the World Health Organization (WHO) by two or three standard deviations.	
Percentage of children under 5 years overweight (modeled estimate) (%)	Proportion of children in this age group whose weight-for-height is above +2 standard deviations (SD) compared to the median of the child growth references established by the WHO.	
Prevalence of low birth weight (%)	Weight below the median of the reference population adopted by the World Health Organization (WHO) by two standard deviations (moderate underweight) or below the median by three standard deviations (severe underweight).	
Average fat supply (g/cap/day) (3-year average)	Amount of fat in food, in grams per day, available for each person of the total population during the reference period. Fat content is determined by applying appropriate food composition factors to the amounts of products. The supply per person is calculated by dividing the total fats by the total population sharing the food during the reference period. However, per-person values represent only the average supply available to the entire population and do not strictly indicate individual consumption. Real food consumption may be lower due to food loss and waste during storage, preparation, cooking, leftovers, or disposal.	Food and Agriculture Organization of the United Nations (FAO) [34] and IHME (the Institute for Health Metrics and Evaluation) and 2019 Global Burden of Disease (GBD) [35]
Seafood, fat supply (g/person/day)		
Milk and dairy products, fat supply (g/person/day)		
Eggs and their products, fat supply (g/person/day)		
Sweets and sugars, fat supply (g/person/day)		
Fats and oils, energy supply (kcal/person/day)	Amount of fat in food, in grams per day, available for each person of the total population during the reference period. Fat content is determined by applying appropriate food composition factors to the amounts of products. The supply per person is calculated by dividing the total fats by the total population sharing the food during the reference period. However, per-person values represent only the average supply available to the entire population and do not strictly indicate individual consumption. Real food consumption may be lower due to food loss and waste during storage, preparation, cooking, leftovers, or disposal.	Food and Agriculture Organization of the United Nations (FAO) [34]
Low seafood omega-3 fatty acid consumption	Defined as average daily consumption (in milligrams per day) of less than 470–660 milligrams of eicosapentaenoic acid (EPA) and docosahexaenoic acid (DHA).	Food and Agriculture Organization of the United Nations (FAO) [34]
n-6 polyunsaturated fatty acid consumption *	Defined as average daily consumption (in % daily energy) of less than 9–10% total energy intake from omega-6, specifically linoleic acid, γ-linolenic acid, eicosadienoic acid, dihomo-γ-linolenic acid, and arachidonic acid.	Food and Agriculture Organization of the United Nations (FAO) [34]
Diet in trans fatty acids	Exposure to a diet in trans fatty acids across all age groups in 2019 is presented as the rate of exposure per 100 individuals. Defined as intake greater than 0–1.1% daily energy of trans fat from all sources, mainly partially hydrogenated vegetable oils and ruminant products.	Food and Agriculture Organization of the United Nations (FAO) [34]

The data correspond to the period from 2019 to 2023 and were obtained from international databases, including FAO and WHO. These estimates cover 183 countries and provide a global perspective on the analyzed variables. Values are averages and may not capture specific variations within each country. * Values represent the average daily consumption of n-6 polyunsaturated fatty acids (e.g., linoleic acid, γ-linolenic acid, eicosadienoic acid, dihomo-γ-linolenic acid, and arachidonic acid) as a percentage of total energy intake. Data are based on the FAO’s definition of n-6 fatty acid consumption, not total polyunsaturated fatty acid intake.

**Table 2:** Predictive variables for the incidence rate of cardiovascular diseases: age-standardized analysis for both sexes.

Variable	AUC	95%CI	*p*-Value	Cut Off Value	SEN%	SPEC%	PPV%	NPV%
Prevalence of low birth weight (%)	0.606	0.512–0.700	0.0270	0.750	65.80	58.90	61.60	63.30
Polyunsaturated fatty acid consumption	0.650	0.566–0.734	<0.0001	0.650	86.70	43.20	61.00	76.00
Low seafood omega-3 fatty acid consumption	0.676	0.591–0.760	<0.0001	0.650	89.30	53.80	67.00	82.70
Diet in trans fatty acids	0.462	0.365–0.559	0.4020	0.650	55.70	87.10	80.00	67.90
Prevalence of undernourishment (%)	0.675	0.588–0.761	<0.0001	0.650	87.50	57.30	68.60	81.10
Political stability and absence of violence or food terrorism (index)	0.351	0.272–0.430	0.0021	0.650	62.50	84.00	78.60	70.50
Average fat supply (g/cap/day) (3-year average)	0.409	0.320–0.497	0.0390	0.650	61.00	87.80	82.00	71.20

AUC (area under the curve), SEN (sensitivity), SPEC (specificity), PPV (positive predictive value), and NPV (negative predictive value) are utilized.

**Table 3:** Comparison of the demographic and anthropometric characteristics according to sex and nutritional status.

		Median and Range	*p*-Value *	Healthy Weight +	Obesity +	*p*-Value *
Age (Months)	Girls (*n* = 29)	106 (77–120)	0.170	106 (68–117)	109 (84–123)	0.186
	Boys (*n* = 38)	107 (93–129)		116 (92–129)	106 (93–131)	0.977
	Total children (*n* = 67)	106 (87–125)		106 (73–125)	106 (89–126)	0.526
Height (m)	Girls	1.33 (1.24–1.45)	0.429	1.31 (1.12–1.38)	1.42 (1.32–1.48)	0.026
	Boys	1.39 (1.24–1.45)		1.34 (1.24–1.44)	1.43 (1.32–1.46)	0.096
	Total children	1.36 (125–1.45)		1.32 (1.17–1.39)	1.43 (1.32–1.46)	<0.0001
Weight (kg)	Girls	35.8 (25.6–49.2)	0.305	27.9 (18.5–31.6)	49.2 (38.5–67.3)	<0.0001
	Boys	38.5 (29.3–58.3)		29.4 (23.3–36.7)	53.9 (40.1–60.3)	<0.0001
	Total children	36.9 (28.4–52.7)		28.8 (21.8–35.6)	50 (39.5–61.2)	<0.0001
WC (cm)	Girls	66.5 (55–83)	0.127	55 (53–63)	83 (74–90)	<0.0001
	Boys	72.0 (60–89)		61 (57–70)	89 (75–92)	<0.0001
	Total children	71 (59–86)		59.7 (54.7–66.5)	86 (75–92)	<0.0001
BMI (kg/m^2^)	Girls	18.6 (15.5–24.8)	0.250	15.5 (15.0–17.3)	24.8 (22.3–29.7)	<0.0001
	Boys	21.0 (16.5–27.1)		16.6 (16.0–18.7)	27.1 (23.6–29.0)	<0.0001
	Total children	19.4 (16.2–24.9)		16.2 (15.3–17.7)	24.9 (23.4–29.1)	<0.0001
WHR	Girls	0.50 (0.44–0.58)	0.135	0.44 (0.43–0.48)	0.58 (0.54–0.59)	<0.0001
	Boys	0.52 (0.47–0.62)		0.47 (0.45–0.48)	0.62 (0.57–0.63)	<0.0001
	Total children	0.50 (0.46–0.59)		0.46 (0.44–0.48)	0.59 (0.56–0.63)	<0.0001

All values are presented as median (range). Abbreviations: WC, waist circumference; BMI, body mass index; WHR, waist circumference-to-height ratio. * *p*-values were calculated using the Mann–Whitney U test. + Classification based on the World Health Organization (WHO) 2007 Child Growth Reference (8). Total: Girls (*n* = 29); Boys (*n* = 38).

## Data Availability

The datasets used and/or analyzed during the current study are available from the corresponding author upon reasonable request.

## References

[1] Vollset S.E., Ababneh H.S., Abate Y.H., Abbafati C., Abbasgholizadeh R., Abbasian M., Abbastabar H., Abd Al Magied A.H.A., Abd ElHafeez S., Abdelkader A. (2024). Burden of Disease Scenarios for 204 Countries and Territories, 2022–2050: A Forecasting Analysis for the Global Burden of Disease Study 2021. Lancet.

[2] National Research Council (US) Committee on Diet and Health (1989). Diet and Health: Implications for Reducing Chronic Disease Risk.

[3] Ghosh S., Molcan E., DeCoffe D., Dai C., Gibson D.L. (2013). Diets Rich in *n* -6 PUFA Induce Intestinal Microbial Dysbiosis in Aged Mice. Br. J. Nutr..

[4] Sears C., Ghosh S. (2016). Excess Omega-6 Polyunsaturated Fatty Acid Intake Is Associated with Negative Cardiovascular, Intestinal and Metabolic Outcomes in Mice. Can. J. Diabetes.

[5] Chen W., Zhang S., Hu X., Chen F., Li D. (2023). A Review of Healthy Dietary Choices for Cardiovascular Disease: From Individual Nutrients and Foods to Dietary Patterns. Nutrients.

[6] Clemente-Suárez V.J., Beltrán-Velasco A.I., Redondo-Flórez L., Martín-Rodríguez A., Tornero-Aguilera J.F. (2023). Global Impacts of Western Diet and Its Effects on Metabolism and Health: A Narrative Review. Nutrients.

[7] Abrignani V., Salvo A., Pacinella G., Tuttolomondo A. (2024). The Mediterranean Diet, Its Microbiome Connections, and Cardiovascular Health: A Narrative Review. Int. J. Mol. Sci..

[8] Kromhout D. (1989). Food Consumption Patterns in the Seven Countries Study. Ann. Med..

[9] Shetty P. (2013). Nutrition Transition and Its Health Outcomes. Indian J. Pediatr..

[10] Popkin B.M., Ng S.W. (2022). The Nutrition Transition to a Stage of High Obesity and Noncommunicable Disease Prevalence Dominated by Ultra-processed Foods Is Not Inevitable. Obes. Rev..

[11] Kenar J.A., Moser B.R., List G.R. (2017). Naturally Occurring Fatty Acids. Fatty Acids.

[12] Mercola J., D’Adamo C.R. (2023). Linoleic Acid: A Narrative Review of the Effects of Increased Intake in the Standard American Diet and Associations with Chronic Disease. Nutrients.

[13] Froyen E., Burns-Whitmore B. (2020). The Effects of Linoleic Acid Consumption on Lipid Risk Markers for Cardiovascular Disease in Healthy Individuals: A Review of Human Intervention Trials. Nutrients.

[14] Marangoni F., Agostoni C., Borghi C., Catapano A.L., Cena H., Ghiselli A., La Vecchia C., Lercker G., Manzato E., Pirillo A. (2020). Dietary Linoleic Acid and Human Health: Focus on Cardiovascular and Cardiometabolic Effects. Atherosclerosis.

[15] Einerhand A.W.C., Mi W., Haandrikman A., Sheng X.-Y., Calder P.C. (2023). The Impact of Linoleic Acid on Infant Health in the Absence or Presence of DHA in Infant Formulas. Nutrients.

[16] Dávila-Torres J., González-Izquierdo J.J., Barrera-Cruz A. (2015). Obesity in Mexico. Rev. Med. Inst. Mex. Seguro Soc..

[17] Shamah-Levy T., Cuevas-Nasu L., Gaona-Pineda E.B., Valenzuela-Bravo D.G., Méndez Gómez-Humarán I., Ávila-Arcos M.A. (2022). Childhood Obesity in Mexico: Influencing Factors and Prevention Strategies. Front. Public Health.

[18] Khandelwal S., Kelly L., Malik R., Prabhakaran D., Reddy S. (2013). Impact of Omega-6 Fatty Acids on Cardiovascular Outcomes: A Review. J. Prev. Cardiol..

[19] Oppedisano F., Macrì R., Gliozzi M., Musolino V., Carresi C., Maiuolo J., Bosco F., Nucera S., Caterina Zito M., Guarnieri L. (2020). The Anti-Inflammatory and Antioxidant Properties of n-3 PUFAs: Their Role in Cardiovascular Protection. Biomedicines.

[20] Jang H., Park K. (2020). Omega-3 and Omega-6 Polyunsaturated Fatty Acids and Metabolic Syndrome: A Systematic Review and Meta-Analysis. Clin. Nutr..

[21] Al-Shaer A.E., Buddenbaum N., Shaikh S.R. (2021). Polyunsaturated Fatty Acids, Specialized pro-Resolving Mediators, and Targeting Inflammation Resolution in the Age of Precision Nutrition. Biochim. Et Biophys. Acta (BBA) Mol. Cell Biol. Lipids.

[22] Gonzalez-Becerra K., Barron-Cabrera E., Muñoz-Valle J.F., Torres-Castillo N., Rivera-Valdes J.J., Rodriguez-Echevarria R., Martinez-Lopez E. (2023). A Balanced Dietary Ratio of N-6:N-3 Polyunsaturated Fatty Acids Exerts an Effect on Total Fatty Acid Profile in RBCs and Inflammatory Markers in Subjects with Obesity. Healthcare.

[23] Bishehkolaei M., Pathak Y. (2024). Influence of Omega N-6/n-3 Ratio on Cardiovascular Disease and Nutritional Interventions. Hum. Nutr. Metab..

[24] Maki K.C., Eren F., Cassens M.E., Dicklin M.R., Davidson M.H. (2018). ω-6 Polyunsaturated Fatty Acids and Cardiometabolic Health: Current Evidence, Controversies, and Research Gaps. Adv. Nutr..

[25] Czernichow S., Thomas D., Bruckert E. (2010). N-6 Fatty Acids and Cardiovascular Health: A Review of the Evidence for Dietary Intake Recommendations. Br. J. Nutr..

[26] Poli A., Agostoni C., Visioli F. (2023). Dietary Fatty Acids and Inflammation: Focus on the n-6 Series. Int. J. Mol. Sci..

[27] Lister N.B., Baur L.A., Felix J.F., Hill A.J., Marcus C., Reinehr T., Summerbell C., Wabitsch M. (2023). Child and Adolescent Obesity. Nat. Rev. Dis. Primers.

[28] Del Río-Navarro B.E., Velázquez-Monroy O., Sánchez-Castillo C.P., Lara-Esqueda A., Berber A., Fanghänel G., Violante R., Tapia-Conyer R., James W.P.T. (2004). The High Prevalence of Overweight and Obesity in Mexican Children. Obes. Res..

[29] Arellano-Alvarez P., Muñoz-Guerrero B., Ruiz-Barranco A., Garibay-Nieto N., Hernandez-Lopez A.M., Aguilar-Cuarto K., Pedraza-Escudero K., Fuentes-Corona Z., Villanueva-Ortega E. (2023). Barriers in the Management of Obesity in Mexican Children and Adolescents through the COVID-19 Lockdown—Lessons Learned and Perspectives for the Future. Nutrients.

[30] Sanders T.A.B. (2019). Omega-6 Fatty Acids and Cardiovascular Disease. Circulation.

[31] Savva S., Tornaritis M., Savva M., Kourides Y., Panagi A., Silikiotou N., Georgiou C., Kafatos A. (2000). Waist Circumference and Waist-to-Height Ratio Are Better Predictors of Cardiovascular Disease Risk Factors in Children than Body Mass Index. Int. J. Obes..

[32] Pérez-Romero S., Gascón-Cánovas J.J., Salmerón-Martínez D., Parra-Hidalgo P., Monteagudo-Piqueras O. (2016). Características Sociodemográficas y Variabilidad Geográfica Relacionada Con La Satisfacción Del Paciente En Atención Primaria. Rev. De Calid. Asist..

[33] Ritchie H., Roser M. (2022). Alcohol Consumption. Our World in Data.

[34] Food and Agriculture Organization of the United Nations FAOSTAT Data. https://www.fao.org/faostat/en/#data.

[35] Institute for Health Metrics and Evaluation Global Burden of Disease (GBD). Study Compare. https://vizhub.healthdata.org/gbd-compare/#.

[36] Farfán Gutiérrez M., Pérez-Salicrup D.R., Flamenco-Sandoval A., Nicasio-Arzeta S., Mas J.-F., Ramírez Ramírez I. (2018). Modeling Anthropic Factors as Drivers of Wildfire Occurrence at the Monarch Butterfly Biosphere. Madera Bosques.

[37] Li H., Song X., Liang Y., Bai X., Liu-Huo W.-S., Tang C., Chen W., Zhao L. (2022). Global, Regional, and National Burden of Disease Study of Atrial Fibrillation/Flutter, 1990–2019: Results from a Global Burden of Disease Study, 2019. BMC Public Health.

[38] Hintermeier M., Gold A.W., Erdmann S., Perplies C., Bozorgmehr K., Biddle L. (2022). From Research into Practice: Converting Epidemiological Data into Relevant Information for Planning of Regional Health Services for Refugees in Germany. Int. J. Environ. Res. Public Health.

[39] Dudley W.N., Benuzillo J.G., Carrico M.S. (2004). SPSS and SAS Programming for the Testing of Mediation Models. Nurs. Res..

[40] Haynes W. (2013). Benjamini–Hochberg Method. Encyclopedia of Systems Biology.

[41] Hu J.X., Zhao H., Zhou H.H. (2010). False Discovery Rate Control with Groups. J. Am. Stat. Assoc..

[42] Polo T.C.F., Miot H.A. (2020). Aplicações Da Curva ROC Em Estudos Clínicos e Experimentais. J. Vasc. Bras..

[43] Safari S., Baratloo A., Elfil M., Negida A. (2016). Evidence Based Emergency Medicine; Part 5 Receiver Operating Curve and Area under the Curve. Emergency.

[44] Unal I. (2017). Defining an Optimal Cut-Point Value in ROC Analysis: An Alternative Approach. Comput. Math. Methods Med..

[45] Evans H.J., Gibson N.A., Bennett J., Chan S.Y., Gavlak J., Harman K., Ismail-Koch H., Kingshott R.N., Langley R., Morley A. (2023). British Thoracic Society Guideline for Diagnosing and Monitoring Paediatric Sleep-Disordered Breathing. Thorax.

[46] Stevens G.A., Alkema L., Black R.E., Boerma J.T., Collins G.S., Ezzati M., Grove J.T., Hogan D.R., Hogan M.C., Horton R. (2016). Guidelines for Accurate and Transparent Health Estimates Reporting: The GATHER Statement. PLoS Med..

[47] Flores R., Telles E. (2012). Social Stratification in Mexico. Am. Sociol Rev..

[48] Luquín-García M.D., Macedo Ruíz E.C., Rojas-Altamirano O., López-Hernández C. (2018). Determination of the Representative Socioeconomic Level by BSA in the Mexican Republic. Rev. Perspect. Empres..

[49] Nayeri H., Naderi G.A., Asgari S., Sadeghi M., Boshtam M., Mohamadzadeh S., Babaknejad N. (2017). LDL Fatty Acids Composition as a Risk Biomarker of Cardiovascular Disease. Artery Res..

[50] Folch J., Lees M., Stanley G.H.S. (1957). A simple method for the isolation and purification of total lipides from animal tissues. J. Biol. Chem..

[51] Eggers L.F., Schwudke D. (2016). Liquid Extraction: Folch. Encyclopedia of Lipidomics.

[52] Rosner B. (2010). Fundamentals of Biostatistics.

[53] ClinCalc.com Statistics. Post-hoc Power Calculator Post-Hoc Power Calculator. Evaluate Statistical Power of an Existing Study. https://clincalc.com/stats/Power.aspx.

[54] World Medical Association (2013). World Medical Association Declaration of Helsinki: Ethical principles for medical research involving human subjects. JAMA.

[55] Kurotani K., Karunapema P., Jayaratne K., Sato M., Hayashi T., Kajio H., Fukuda S., Hara H., Okazaki O., Jayatilleke A.U. (2018). Circulating Odd-Chain Saturated Fatty Acids Were Associated with Arteriosclerosis among Patients with Diabetes, Dyslipidemia, or Hypertension in Sri Lanka but Not Japan. Nutr. Res..

[56] Poudyal H., Kumar S.A., Iyer A., Waanders J., Ward L.C., Brown L. (2013). Responses to Oleic, Linoleic and α-Linolenic Acids in High-Carbohydrate, High-Fat Diet-Induced Metabolic Syndrome in Rats. J. Nutr. Biochem..

[57] Mousavi S.M., Jalilpiran Y., Karimi E., Aune D., Larijani B., Mozaffarian D., Willett W.C., Esmaillzadeh A. (2021). Dietary Intake of Linoleic Acid, Its Concentrations, and the Risk of Type 2 Diabetes: A Systematic Review and Dose-Response Meta-Analysis of Prospective Cohort Studies. Diabetes Care.

[58] Raatz S.K., Conrad Z., Jahns L. (2018). Trends in Linoleic Acid Intake in the United States Adult Population: NHANES 1999–2014. Prostaglandins Leukot. Essent. Fat. Acids.

[59] Marklund M., Wu J.H.Y., Imamura F., Del Gobbo L.C., Fretts A., de Goede J., Shi P., Tintle N., Wennberg M., Aslibekyan S. (2019). Biomarkers of Dietary Omega-6 Fatty Acids and Incident Cardiovascular Disease and Mortality. Circulation.

[60] Aceves-Martins M., López-Cruz L., García-Botello M., Gutierrez-Gómez Y.Y., Moreno-García C.F. (2022). Interventions to Treat Obesity in Mexican Children and Adolescents: Systematic Review and Meta-Analysis. Nutr. Rev..

[61] Turnbull B., Gordon S.F., Martínez-Andrade G.O., González-Unzaga M. (2019). Childhood Obesity in Mexico: A Critical Analysis of the Environmental Factors, Behaviours and Discourses Contributing to the Epidemic. Health Psychol. Open.

[62] Mercado-Mercado G. (2023). Childhood Obesity in Mexico: A Constant Struggle and Reflection for Its Prevention on the Influence of Family and Social Habits. Obes. Med..

[63] Díaz-Urbina D., Escartín-Pérez R.E., López-Alonso V.E., Mancilla-Díaz J.M. (2018). Efectos de Una Dieta Con Alto Contenido de Grasas Sobre Patrones Conductuales Alimentarios. Acta Colomb. Psicol..

[64] Fekete K., Györei E., Lohner S., Verduci E., Agostoni C., Decsi T. (2015). Long-chain Polyunsaturated Fatty Acid Status in Obesity: A Systematic Review and Meta-analysis. Obes. Rev..

[65] Balas-Nakash M., Villanueva-Quintana A., Tawil-Dayan S., Schiffman-Selechnik E., Suverza-Fernández A., Vadillo-Ortega F., Perichart-Perera O. (2008). Studio Piloto Para La Identificación de Indicadores Antropométricos Asociados a Marcadores de Riesgo de Síndrome Metabólico En Escolares Mexicanos. Bol. Med. Hosp. Infant. Mex..

[66] Morandi A., Miraglia del Giudice E., Martino F., Martino E., Bozzola M., Maffeis C. (2014). Anthropometric Indices Are Not Satisfactory Predictors of Metabolic Comorbidities in Obese Children and Adolescents. J. Pediatr..

[67] Mehta S.K. (2016). Waist Circumference to Height Ratio and Left Ventricular Mass in Children and Adolescents. Cardiol. Young.

[68] Barbieri A., Bursi F., Mantovani F., Valenti C., Quaglia M., Berti E., Marino M., Modena M.G. (2011). Prognostic Impact of Left Ventricular Mass Severity According to the Classification Proposed by the American Society of Echocardiography/European Association of Echocardiography. J. Am. Soc. Echocardiogr..

[69] Maffeis C., Banzato C., Talamini G. (2008). Waist-to-Height Ratio, a Useful Index to Identify High Metabolic Risk in Overweight Children. J. Pediatr..

[70] Moreira Andrés M.N. (2010). ¿Qué Medida Antropométrica de Exceso de Peso Discrimina Mejor El Riesgo Cardiovascular?. Med. Clin..

[71] Adolph T.E., Tilg H. (2024). Western Diets and Chronic Diseases. Nat. Med..

[72] Smolińska K., Szopa A., Sobczyński J., Serefko A., Dobrowolski P. (2024). Nutritional Quality Implications: Exploring the Impact of a Fatty Acid-Rich Diet on Central Nervous System Development. Nutrients.

[73] Simopoulos A.P., DiNicolantonio J.J. (2016). The Importance of a Balanced ω-6 to ω-3 Ratio in the Prevention and Management of Obesity. Open Heart.

[74] Hodson L., Skeaff C.M., Fielding B.A. (2008). Fatty Acid Composition of Adipose Tissue and Blood in Humans and Its Use as a Biomarker of Dietary Intake. Prog. Lipid Res..

[75] Katan M.B., Deslypere J.P., van Birgelen A.P., Penders M., Zegwaard M. (1997). Kinetics of the Incorporation of Dietary Fatty Acids into Serum Cholesteryl Esters, Erythrocyte Membranes, and Adipose Tissue: An 18-Month Controlled Study. J. Lipid Res..

